# Radiologic Evaluation Of Lumbar Spinal Stenosis: The Integration Of Sagittal And Axial Views In Decision Making For Minimally Invasive Surgical Procedures

**DOI:** 10.7759/cureus.4268

**Published:** 2019-03-19

**Authors:** Jason Hartman, Michelle Granville, Robert E Jacobson

**Affiliations:** 1 Pain Medicine, Larkin Community Hospital, Miami, USA; 2 Neurological Surgery, University of Miami Hospital, Miami, USA

**Keywords:** lumbar spinal stenosis, neurogenic claudication, low back pain

## Abstract

Radiologic findings in combination with clinical symptoms are critical in the diagnosis and evaluation of the severity of lumbar spinal stenosis (LSS) as well as the need for surgical treatment. Dynamic radiographs, computerized tomography (CT), and magnetic resonance imaging (MRI) each provide different but interrelated pieces of information in the patient with lumbar spinal stenosis. Making a treatment decision based only on one of the radiographic studies may negatively affect the treatment outcome. Minimal procedures are predicated on identifying and performing surgery on a limited segment of the lumbar spinal canal affected by the stenosis compared to what occurs during open surgery where the judgment of the spine surgeon often expanded the decompression area based on real-time intra-operative findings correlated with radiologic findings of stenosis. As newer, less invasive procedures are gaining acceptance for surgical treatment of spinal stenosis with symptomatic claudication, radiologic studies become more critical in selecting the correct procedure since there may be no or minimal surgical visual confirmation of the pathology. This article will review how the finding of spinal deformity and motion, canal dimensions, viewed in multiple planes and the presence of facet fluid impact treatment decisions. Differences in these abnormal radiologic findings can affect the selection of surgical procedures ranging from open decompression with pedicle fixation, decompression with interlaminar stabilization, minimally invasive lumbar decompression, and percutaneous interspinous implants providing distraction without decompression. With the development of less invasive procedures, lumbar spinal stenosis is being evaluated and treated not only by spine surgeons but also by interventional pain and neuroradiology physicians that may not be totally familiar with the complexity of the pathology and neuro-radiology of LSS. Each radiologic study provides different information. The goal of this report is to provide a framework for the use of studies such as plain X-rays, dynamic films, MRI, and CT scans as well as the importance of different views, and how to use them in evaluating the abnormal radiologic anatomy seen with LSS and in selecting the most appropriate procedure.

## Introduction

Many older patients are found to have radiologic lumbar spinal stenosis (LSS) when examining magnetic resonance imaging (MRI) and computerized tomography (CT) scans of the lumbar spine, but are asymptomatic or have only minimal low back pain without evidence of progressive neurogenic claudication (NC), the hallmark of clinical symptomatology with LSS [1-2]. Paradoxically, patients with symptomatic neurogenic claudication are often found to have very similar radiologic findings to the asymptomatic patient with radiologic evidence of LSS, which indicates that there is not a simple direct relationship between the presence and degree of radiologic narrowing of the lumbar spinal canal seen on MRI and CT scans and clinical symptoms of neurogenic claudication [3]. There are multiple etiologies for LSS, including congenital short pedicles, degenerative spondylolisthesis, with or without instability, one level or multi-segmental spinal degeneration secondary to facet hypertrophy and adjacent segment degeneration, and stenosis above previous lumbar instrumentation and fusion [2-3]. Initially, stenosis was thought to be due to bony narrowing of the spinal canal secondary to congenitally short pedicles or facet bone overgrowth, but the development of axial imaging, CT scanning and especially MRI scans clearly demonstrated that in many cases the stenosis is primarily due to posterior soft tissue ligamentous compression [2-3]. Understanding the information obtained from different radiologic studies helps determine the location, degree and whether the lumbar spinal stenosis is due to bone compression or ligamentous soft tissue. Interpreting radiologic studies and correlating them with clinical symptoms is a critical step in deciding whether and which surgical procedure is necessary. 

## Technical report

Radiology

For purposes of radiologic description and classification, the lumbar canal is divided into sections in both the axial and sagittal spinal planes. The axial, or transverse plane, includes the central canal, lateral recesses and neural foramina, while the vertical or sagittal plane includes the supra-pedicular, pedicular and infra-pedicular regions at each vertebral segment [3]. The pedicular region is relatively fixed, based on the depth of the pedicle, while both the supra-pedicular and infra-pedicular region are affected by ventral disc herniation, vertebral osteophytes, and by medial enlargement of the facet joints, joint capsule, and ligamentum flavum which all contribute to narrowing of the lumbar spinal segment and leads to lumbar stenosis and specifically lateral recess stenosis [4]. Comparing equivalent, same level, images on CT and MRI scans helps differentiate spinal stenosis secondary to bone and facet overgrowth from ligamentous hypertrophy. The CT scan is excellent at delineating bone changes and bony canal size while the MRI shows soft tissue and dura very well. The difference in the dimensions between the two studies shows if compression is due to bone or soft tissue compression or both. LSS is also associated with spinal segmental instability and degenerative spondylolisthesis in 20%-30% of cases, so dynamic plain X-rays are important in the evaluation of the patient, since motion or instability can worsen with flexion and extension and will affect surgical planning and if spinal stabilization is needed [2-3, 5-6]. Plain X-rays, including dynamic standing, flexion and extension films are still critical in preoperative assessment because segmental instability can be missed with supine MRI scans in up to 20% of cases [5, 7]. The finding of segmental motion, usually indicated by sagittal plane movement with flexion/extension, correlates with the degree of abnormal spinal instability and is most commonly found with pre-existing degenerative spondylolisthesis [8]. In many patients with spinal instability, a related and important finding in reviewing MRI scans is the presence of facet joint fluid primarily on axial images and coexists with spinal instability [3]. The presence of spinal instability can change the type of surgical procedures selected when treating neurogenic claudication. Other spinal deformities such as lumbar scoliosis, especially if there is greater than 10% angulation, can cause lateral recess and foraminal stenosis on the concave side of the scoliosis as well as complicate the use of different implants, especially if the scoliosis extends over several segments. Imaging in the sagittal plane, particularly on MRI scan, is the most common way to assess the number of levels of canal narrowing, but the sagittal view alone can underestimate the degree of lateral recess stenosis. The axial plane, using both CT and MRI scan, is the best plane to identify central versus lateral recess stenosis [3-4, 8]. Vertebral osteoporosis is frequently found in the same elderly population that has LSS and is an additional factor that must be considered since insufficient bone strength can be a contraindication for use of both screw implants and even interspinous implants with the risk of spinous process fractures. In these cases, a bone mineral density (BMD) should be performed as part of the clinical evaluation. If the BMD reveals significant osteoporosis, bi-cortical screws and if appropriate, interlaminar or interspinous implants may be a better option for a one level stenosis than pedicle screws.

Assessing severity of lumbar spinal stenosis

The radiologic degree or severity of lumbar stenosis is typically described either qualitatively, as a percentage of narrowing, or quantitatively, by actual measurements in millimeters (mm). Either of the sagittal and coronal dimensions or total canal area are measured in square centimeters (cm^2^) [4-5]. Typically 'mild' to 'moderate' stenosis is described as less than 25% to 50% narrowing while 'severe' stenosis as greater than 50%. A possible explanation given of why patients with greater than 50% central or lateral stenosis can be asymptomatic is that the 'symptoms' when intermittent may be due to root inflammation and vascular compromise rather than simple anatomic compression [5-6, 9]. The effectiveness of epidural steroids and physical therapy lends some support to this hypothesis [5]. In patients with LSS, it is important to compare the reduced canal area, the sagittal length of narrowing, and the spinal segments above and below the compression and determine if the dural sac and nerve roots are compressed gradually over several segments, or very locally at one specific area. A typical example is a patient with L4-L5 stenosis associated with degenerative spondylolisthesis. In this case, both the infra-pedicular level of the upper vertebrae, which would be L4 and the supra-pedicular segment of the lower vertebrae L5, are the areas of severe narrowing secondary to hypertrophy of the L4 and L5 facet joint. Most importantly, stenosis is often not a single plane pathology so it is critical to understand that narrowing not only affects the axial area, but overall spinal canal volume over the entire length of the stenosis. It is important to evaluate and compare the volume changes between the bone and ligaments and the dural sac over multiple slices on MRI and CT scans. Using a combination of various X-rays, CT and MRI findings, it can be difficult to relate the narrowing to clinical symptoms which can vary from localized back pain, unilateral radicular pain, or neurogenic claudication. The treating physician can use provocative tests and questionnaires such as the symptomatic walking index, Oswestry Disability Index (ODI) and the Zurich Claudication Questionnaire (ZCQ) to evaluate the clinical impact of the stenosis as well as the necessity for surgical interventional treatment of any kind [9].

Relationship of radiologic findings to clinical symptoms and neurogenic claudication

With the development and acceptance of more minimal and indirect means of decompression and stabilization for lumbar stenosis such as Coflex^R^ (Paradigm Spine, New York, NY, USA), and as the newer less invasive procedures such as Vertiflex^R^ (Superion, Carlsbad, California, USA ) and Minuteman^R^ ( Spinal Simplicity,Overland Park, KS, USA), it is important to understand the selection based on radiologic degree of stenosis and clinical symptoms. If a patient only has localized back pain or mild lateralized symptoms without neurologic findings or neurogenic claudication, and does not have confirmatory elevated scores on the ZCQ, surgically invasive procedures may not be indicated, but rather physical therapy or epidural steroid blocks. A minimum of six months of conservative treatment is the norm before surgical intervention is considered [10-14]. Different physicians are now being trained to perform procedures for LSS including interventional pain physicians and neuro-interventional radiologists who may not be as aware of the significance of the different radiologic findings in LSS, the evaluation of instability, and clinical variables and how the clinical testing must be integrated in evaluating patients with LSS before any surgical procedure is considered or performed.

Radiologic findings in lumbar stenosis

It is important for the treating physician, before considering any interventional procedure, to review and correlate the information provided by all the different spinal examinations including plain radiographs, dynamic flexion and extension films as well as lateral bending radiographs, reconstructed three-plane CT scans and MRI scans with different images such as T1, T2 and STIR images. Dynamic films show evidence of instability, CT scans show bone detail better, and the MRI provides imaging of the vertebrae, disc space, dura, nerve roots, soft tissue ligaments, and possibly evidence of abnormal fluid in the facet joint. Each study provides different pieces of information, but when they are integrated, they provide a better understanding of the specific pathology as well as the extent and severity of lumbar stenosis in each patient. The examples presented demonstrate the different information and correlation between various images using both dynamic films, CT scans, and MRI scans in the same plane.

Plain Radiographs

**Figure 1:**
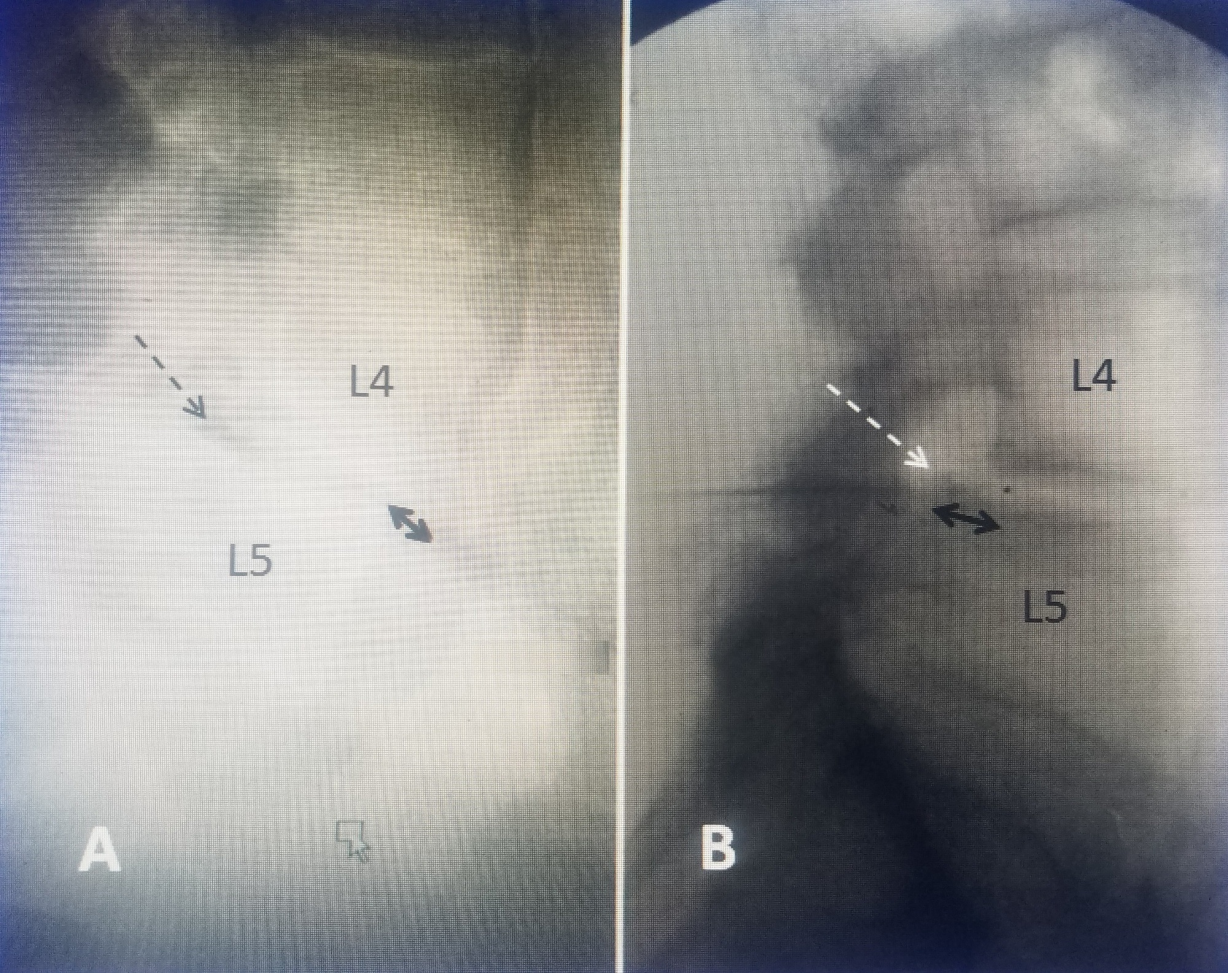
Comparison of different quality lateral views using plain X-rays and digital imaging to evaluate L4-5 spondylolisthesis in the same patient. A: Initial poor quality plain X-rays showing grade 1 spondylolisthesis at L4-5 which is better seen ventrally (double-headed black arrow). The neural foramina can barely be seen (dashed black arrow). B: Better digital radiographic images in the same patient clearly showing the neural foramina (dashed white arrow) as well as the grade 1 spondylolisthesis (double-headed black arrow).

Even in patients with clear clinical symptoms of neurogenic claudication and an MRI showing lumbar stenosis, plain X-rays can show additional information such as spondylolisthesis, scoliosis, congenital abnormalities such as sacralized or lumbarized vertebrae, previous spinal surgery, and especially existence and type of implanted hardware used for previous surgery. However, plain X-rays, if of suboptimal quality or in obese patients, often need to be repeated or reformatted using digital radiography to get better details (Figure 1).

Computerized Tomography

**Figure 2:**
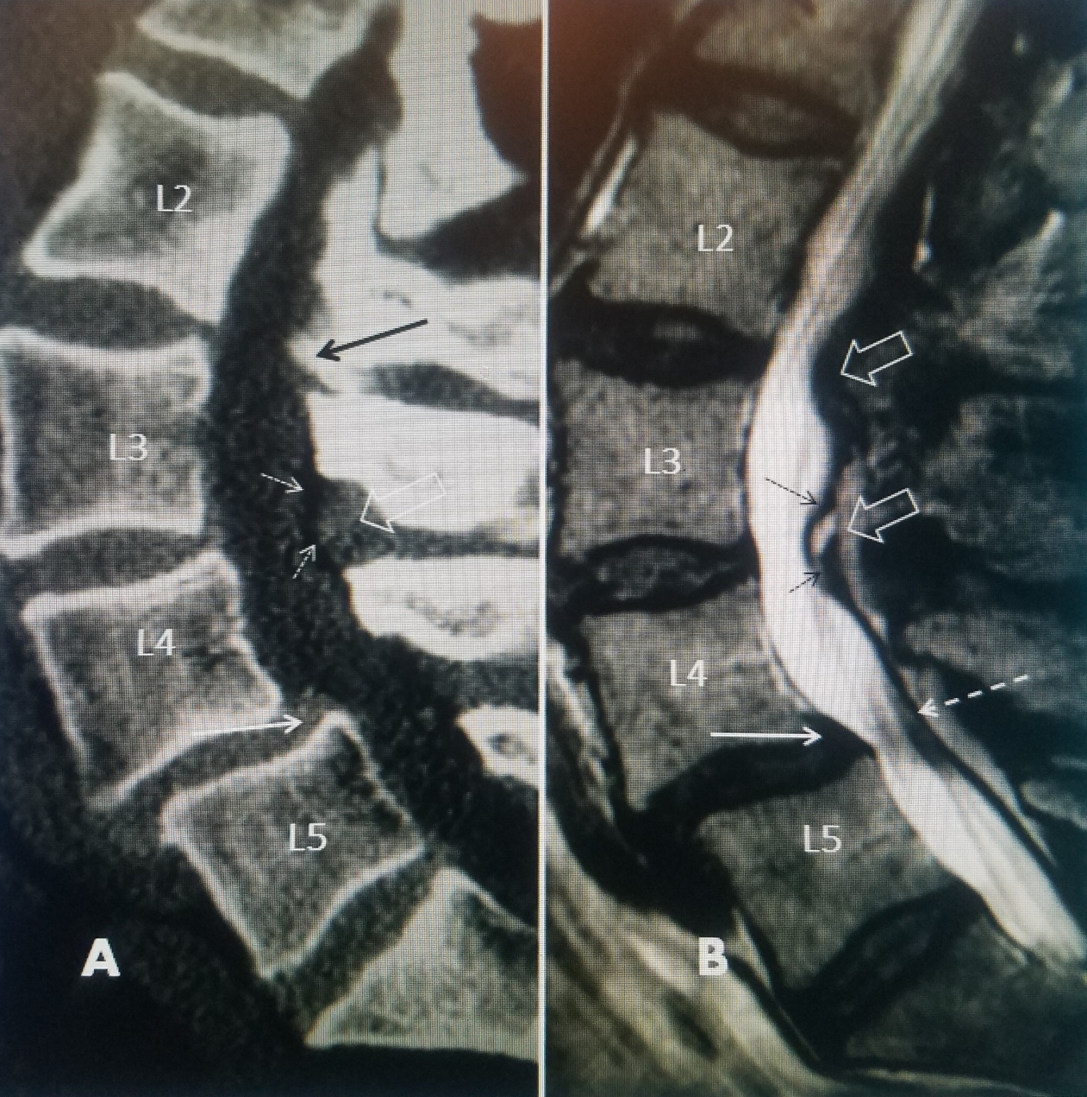
Comparison of computerized tomography (CT) scan and magnetic resonance imaging (MRI) scan in same patient. A: Sagittal CT scan showing L4-5 spondylolisthesis (solid white arrow). At L3-4 there is a mild posterior compression (open white arrow) from ligament hypertrophy into the posterior epidural space (dashed white arrow). There is mild bone compression at L2-3 (solid black arrow). B: Sagittal MRI in the same patient clearly shows there is posterior compression at L4-5 (dashed white arrow) separate from the anteriolisthesis at L4-5 (solid white arrow). The combination of both anterior spondylolisthesis and posterior compression at the same level is causing marked canal narrowing and stenosis at L4-5. The MRI also shows that there is more obvious posterior soft tissue compression at L3-4 (dashed black arrows) as well as additional posterior compression at L2-3 and L3-4 from ligamentous and facet hypertrophy (open white arrows).

CT scans are better in demonstrating bone detail, such as the spinal canal and the facet joints. Soft-tissue compression, especially if posterior from ligamentous hypertrophy or facet cysts, can be identified but should be confirmed with MRI scan primarily in the presence of spondylolisthesis. By comparing similar sections on CT and on MRI scans it can be determined if the sagittal stenosis is due to soft tissue or bone [4, 7]. Image quality and elimination of movement artifact are critical for both CT and MRI scans. Image detail is determined by a combination of slice thickness and overlap, the image matrix for CT scans, and the slice thickness and magnet power for MRI [4, 12]. It is advisable that a minimum of 1.0 Tesla MRI scanner be used when evaluating the spine for surgical planning (Figure 2).

**Figure 3:**
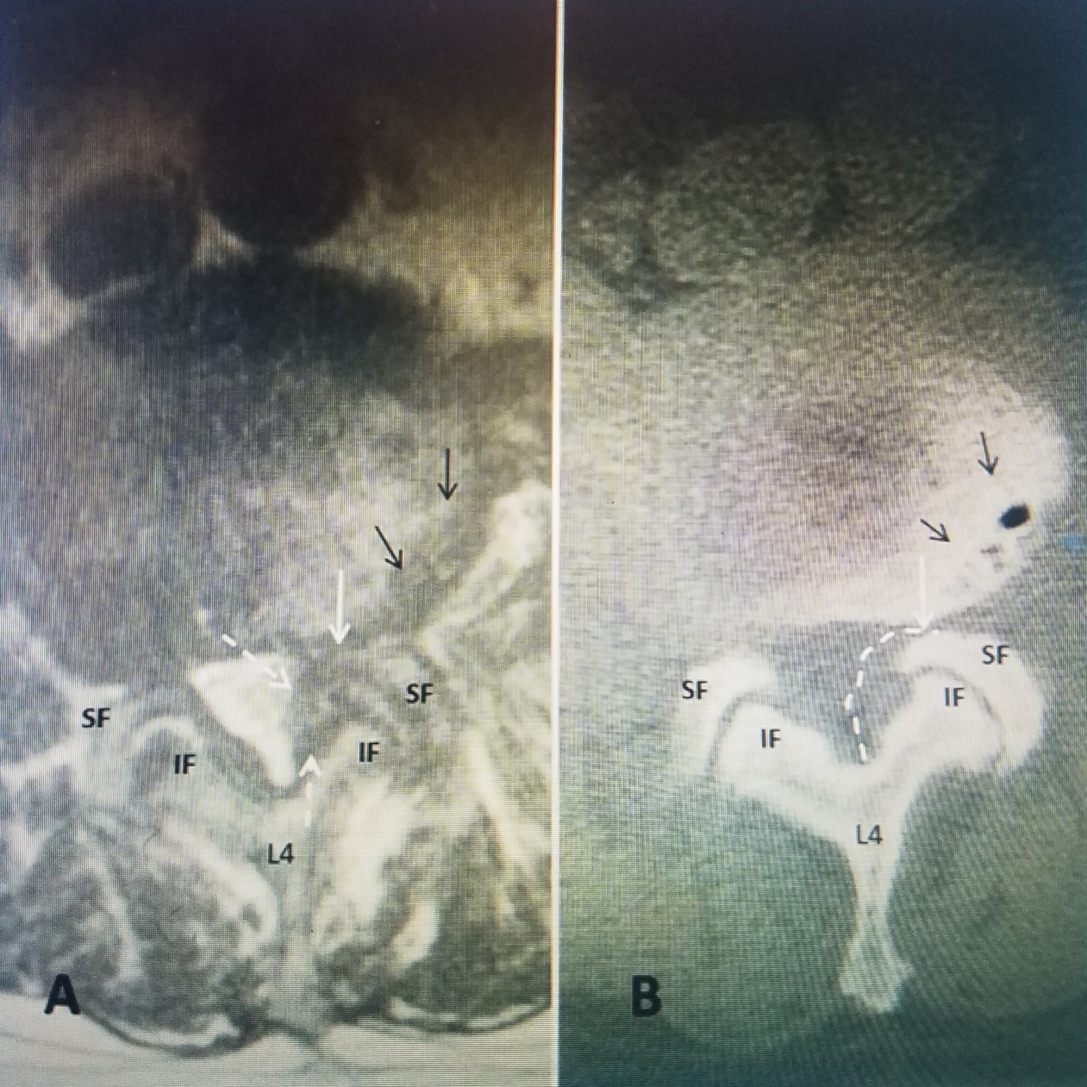
Matched axial cuts at L4-5 in the same patient comparing MRI to CT scan. A: Axial T2 MRI at L4-5 demonstrates the lateral recess stenosis on one side (dashed white arrows) compressing approximately 50% of the lateral spinal canal almost to the midline. The inferior facet (IF) of L4 and superior facet (SF) of L5 are identified. The neural foramina is compressed (solid white arrow) and there are also irregular subchondral endplate cysts in the vertebral body on the same side (solid black arrows). B: Axial CT scan in the same patient made at the same level. Compared to the MRI, the CT scan demonstrates the relationship of the superior facet (SF) to the stenotic foramina (solid white arrow). The soft tissue stenosis seen on MRI in 'A' but not on CT scan is marked(dashed white arrow). The inferior facet (IF) of L4 and the superior facet of L5 can be seen compressing the neural foramina (solid white arrow). Comparing the MRI to the CT demonstrates that the compression seen on the MRI is secondary to soft tissue hypertrophy rather than bony facet overgrowth.

Comparison of the axial views from the MRI to the CT scan helps to delineate if the compression is primarily from soft tissue, causing central or lateral recess stenosis, or due to bone from the hypertrophied facet joints (Figure 3).

Dynamic Radiographs

**Figure 4:**
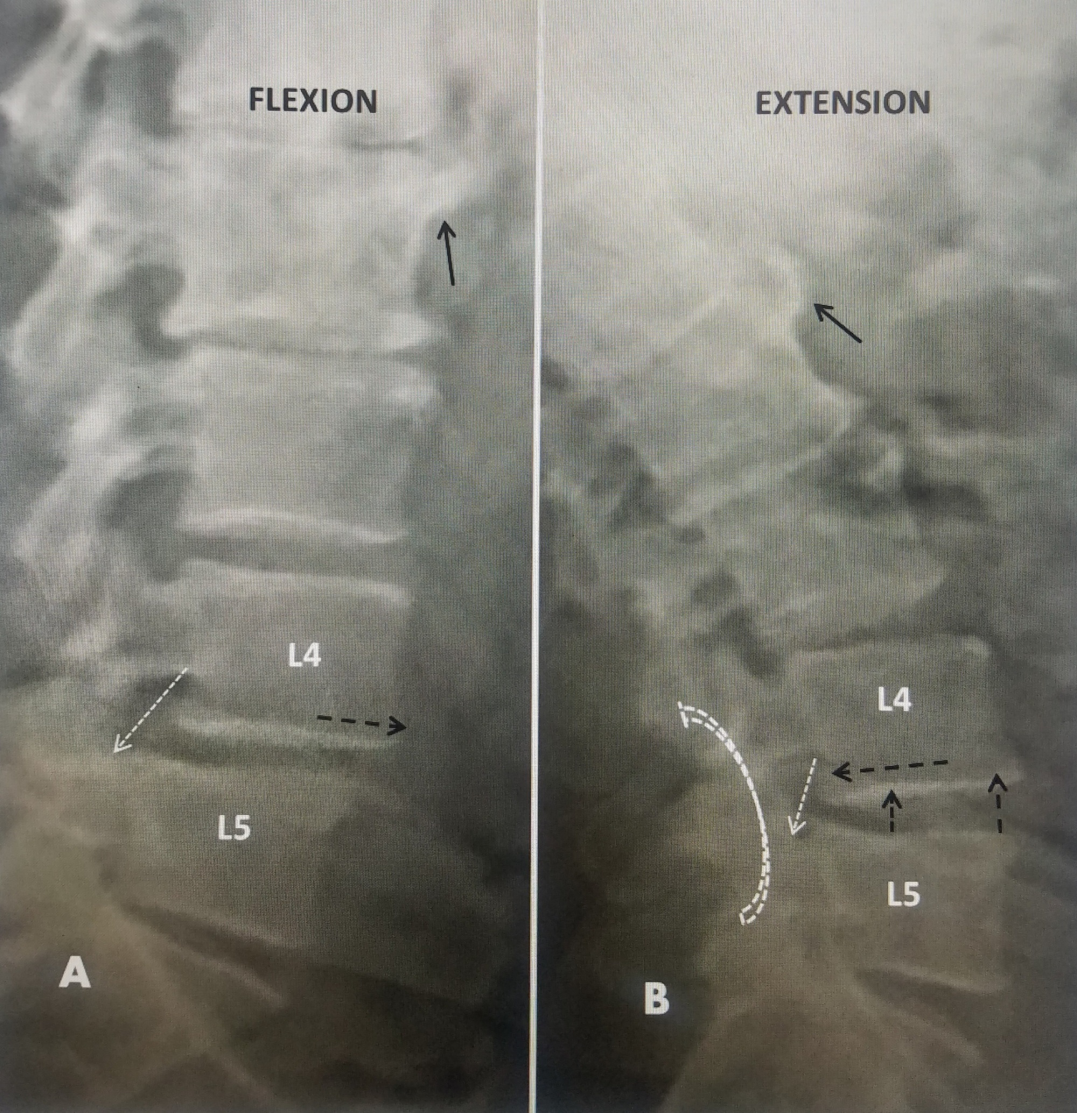
Flexion and extension plain radiographs made to evaluate spinal movement and stability. A: Flexion films show sagittal anterolisthesis at L4-5 (dashed black arrow). The neural foraminal is distorted and mildly reduced in superior-inferior dimension (dashed white arrow). B: Extension films in the same patient shows both posterior rotation (open white curved dashed arrow) and posterior translation of L4 on L5 (dashed black arrow). At the same time the disc space opens anteriorly and narrows posteriorly (solid black arrows). As a result, the neural foramina becomes smaller in extension compared to flexion (solid white arrow) even though the vertebrae are more aligned in extension.

Plain X-rays and especially dynamic lumbar films provide information not necessarily seen on CT or MRI scans. Dynamic films are critical in establishing if there is any changing spinal deformity or movement, the degree of movement in different positions in millimeters and in what direction it occurs. There can be anterolisthesis or retrolisthesis without movement. With movement, the adjacent vertebral endplates can be parallel or assume different angles reflecting different degrees of instability. However, obtaining accurate and reliably consistent dynamic films is often difficult and affected by factors such as patient size, patient movement, and diligence of the radiology technician in obtaining good films [2, 15]. Spinal movement can be both in the sagittal plane or combined with spinal rotation. Radiology criteria for movement is considered anterior-posterior (AP) translation of greater than 5 mm or 50% of the preexisting slip. Narrowing and change to the height of the disc space on lateral views are considered other radiologic findings of segmental instability, especially with angulation of the disc space and change in orientation of the adjacent vertebral endplates [3-4]. In the example illustrated below, there is an unstable L4-5 spondylolisthesis where there is clear forward movement or anterolisthesis in flexion (translation) and then return to alignment in extension. At the same time, the neural foramina gets smaller in extension causing characteristics claudication symptoms. Segmental instability is confirmed by movement with a change in alignment of the vertebral endplates from flexion to extension (Figure 4).

Magnetic Resonance Imaging

**Figure 5:**
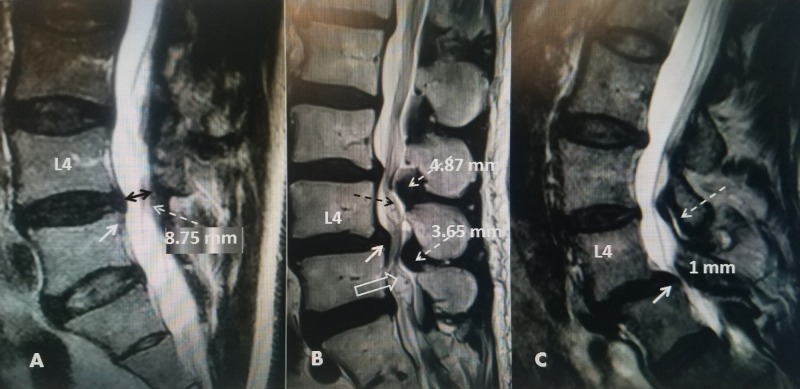
Sagittal T2 MRI scans compared in three different patients with different grades of spinal stenosis. A: Sagittal T2 MRI showing mild stenosis at L4-5 (double-headed black arrow) primarily secondary to ligamentous narrowing of the L4-5 canal posteriorly (dashed white arrow). There is minimal evidence of spondylolisthesis (solid white arrow) but the sagittal canal diameter at L4-5 is reduced to 8.75 mm (dashed white arrow). B: Sagittal midline T2 MRI scan with the MRI plane made directly through the posterior spinous processes showing grade 1 spondylolisthesis at L4-5 (solid white arrow). There is marked posterior ligamentous hypertrophy and encroachment at both L 3-4 and L 4-5 (dashed white arrow). The mid-sagittal canal diameter is 4.87 mm at L3-4 and 3.65 mm at L4-5 (dashed white arrows) The swollen, matted nerve roots (open white arrow) seen just below the L4-5 stenosis are characteristic signs of nerve root edema and compression seen with severe canal stenosis. The swollen cauda equina roots (dashed black arrow) above the stenosis is also characteristic of high-grade stenosis. C: Sagittal T2 MRI showing more severe grade 1-2 spondylolisthesis (solid white arrow) and marked lumbar spinal canal narrowing at L4-5. The sagittal dimension at L4-5 is 1 mm (solid white arrow). There is separate mild L3-4 posterior compression (dashed white arrow).

The MRI scans add further information regarding soft tissue compression. The MRI scan visualizes the vertebral body, spinal canal, facet joint, existence of facet joint fluid, and relationship to the dura, nerve root, and foraminal compression in the lateral recess. Typical MRI signs of root compression can be seen on both sagittal and axial MRI, and includes clumping of the nerve roots in the area of the stenosis, with or without the loss of the cerebrospinal fluid (CSF) signal on both T1 and T2 images and loss off epidural fat signal normally surrounding the dura. These changes can also be apparent in the lateral recesses and neural foramina. There often is associated enlargement, swelling, and a 'serpentine pattern' of the nerve roots of the cauda equina above the stenosis. Other axial MRI findings include dorsal layering or 'gravitation' of the roots with loss of identification of individual rootlets that is typically seen in a normal axial MRI [4-5]. The actual area of the spinal canal in square millimeters more accurately reflects segmental stenosis than sagittal canal dimensions. Although the sagittal plane is valuable for initial identification and comparison, a midline slice can have a false normal depth since the majority of compression may be posterio-lateral secondary to facet and ligamentous hypertrophy. When comparing reconstructed CT scan and MRI in the evaluation of LSS, it is important to understand the marked increase in relative differences between a linear measurement compared to area measurement and then to a volumetric measurement. For example, a normal lumbar canal of 17 mm sagittal x 14 mm mid-facet is 238 mm^2^ or 2.38 cm^2^. A four mm reduction in both planes to 13 x 10 mm, or 24% to 28% linear reduction, becomes an area reduction to 130 mm^2^ or 1.30 cm^2^ which is a 55% reduction in canal area from the normal. If that is converted over the length of the stenosis the volumetric reduction is even more marked. Using both CT and MRI can help distinguish between bone and soft tissue ligamentous overgrowth and compression and also provide an evaluation of what length of the canal is narrowed, which affects the overall volume of spinal canal compression. Although, typically MRI scans are performed with the patient laying down, studies show that in standing MRI scans with spinal extension there may be an additional decrease in spinal lumbar spinal volume from posterior indentation of the ligamentum flavum and the facet joints [5-7] (figure 5).

Making measurements for comparison of sagittal and axial imaging

**Figure 6:**
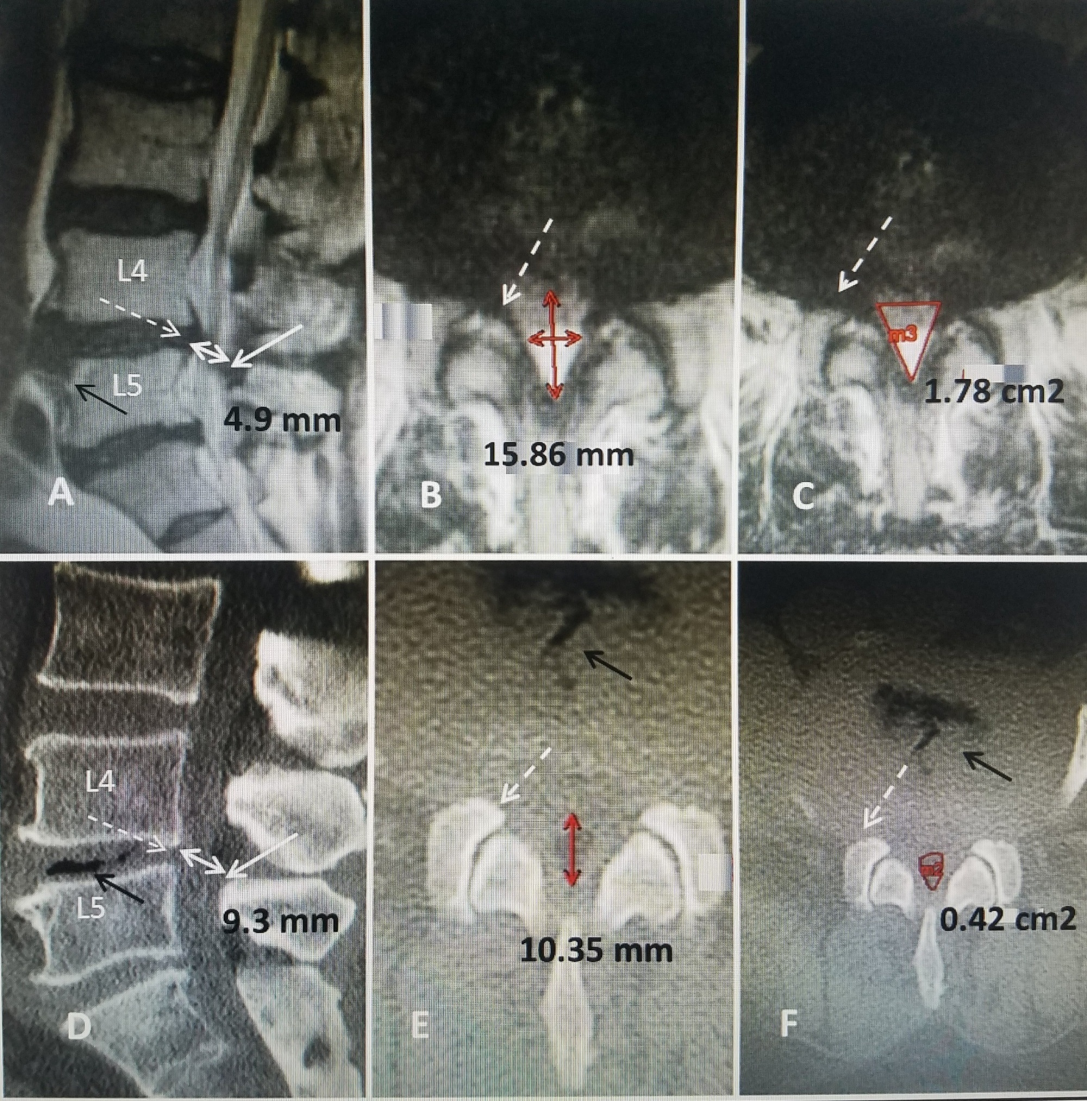
Lumbar canal measurements comparing both MRI and CT scans in the same planes in the same patient with L4-5 stenosis. A: Sagittal T2 MRI showing L4-5 compression from combination of a ventral herniated disc (dashed white line) and posterior ligamentous compression (solid white arrow). The anterior-posterior canal dimension is 4.9 mm (double-headed white arrow). B; Axial T2 MRI shows a midline sagittal measurement of 15.86 mm. The mid-facet line measurement is 7.22 mm (horizontal solid red arrow). The lateral disc that is seen in 'A' (dashed white arrow). C: Calculating axial area using an area cursor gives canal area of 1.78 cm^2^. The lateral disc is seen at L4-5 (dashed white arrow). D: Sagittal CT scan performed on same patient. The anterior-posterior canal dimension is 9.3 mm (solid white double-headed arrow). The midline sagittal slice is similar to the MRI scan in 'A'. The anterior-posterior canal dimension is 9.3 mm at the L4-5 level. Vacuum changes can be seen within the L4-5 disc space (solid black arrow). Vacuum changes are considered a secondary radiologic sign for both disc degeneration and possibly segmental instability. The difference in anterior-posterior canal dimension from CT to MRI is 4.4 mm, indicating significant soft tissue narrowing. E: Axial CT showing midline soft tissue measurement of 10.35 mm (vertical solid red arrow). There are clear vacuum changes in the disc space (solid black arrow) and lateral foraminal disc compression (dashed white arrow). F: Axial CT measuring the approximate dural area of 0.42 cm^2^ (solid circular red line). This is 24% of the area measured on axial MRI again indicating significant soft tissue rather than bony canal stenosis.

The axial or cross-sectional image is critical in evaluating stenosis and being able to make comparative measurements between CT and MRI images. This helps in determining if the compression is primarily due to bone or soft tissue. Sagittal films can identify the levels and degree of stenosis. Axial imaging provides a better analysis of the central canal and lateral recesses. By using measurements of axial area and using the tools on CT or MRI scans and actual measurements of the bone verse soft tissue spinal canal it is possible to calculate the actual severity of the compression to make surgical decisions and evaluate the patient afterwards (Figure 6).

Another benefit of learning to compare the various planes of CT and MRI is the fact that some elderly patients with symptomatic lumbar spinal stenosis have a pacemaker or neuro-stimulator that prevents them from having an MRI scan. In these patients use of the various measurements as discussed, using the CT scan to measure area or calculate the multiple of the linear sagittal and mid-facet measurement to give a close approximation of spinal canal area in stenosis, can be performed. Some patients can even undergo CT myelography so the difference in area between the bony canal and the dural sac can be more exactly measured. By establishing measurements for comparison as cases are reviewed and 'storing' visual images of stenosis, working with the neuro-radiologist the surgeon can make a reliable radiologic decision if the LSS is due to bone verse ligament hypertrophy [2, 4].

**Figure 7:**
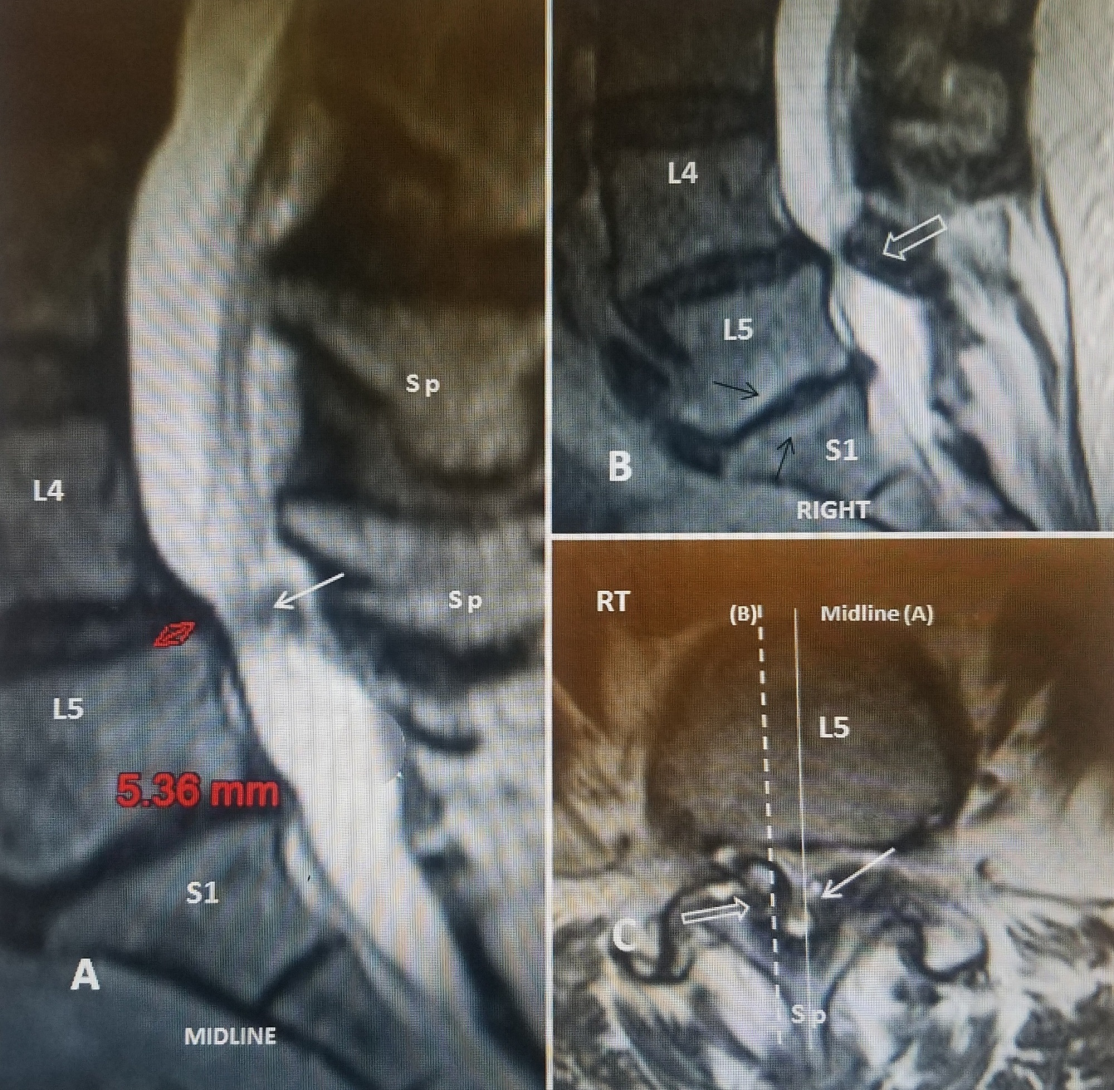
Magnetic resonance imaging demonstrating how to correlate sagittal to axial imaging in lumbar spinal stenosis. A: Midline T2 sagittal slice shows grade 1 spondylolisthesis at L4-5 (solid double-headed red arrow) with a anterior-posterior canal dimension of 5.36 mm. The moderate stenosis seen on this midline slice, identifying by the spinous processes (sp) shows the compression at L4-5 is primarily posteriorly (solid white arrow). B: Sagittal T2 slice made slightly more lateral than the cut in 'A'. The posterior stenosis (open white arrow) now appears more severe than in 'A'. C: Axial T2 MRI in the same patient indicates where each slice is located and the right sided indicated (RT). The midline slice (solid thin white line) including the spinous process (sp), corresponds to 'A'. This is indicated that 'A' is adjacent to the enlarged facet bone and joint (solid white arrow). The more right sided cut (dashed white line) corresponding to 'B' shows the markedly enlarged facet joint and thickened joint capsule and ligament (open white arrow) corresponding to the right lateral recess. In this patient, correlating axial and sagittal MRI scan slices demonstrates the stenosis is worse in the lateral recesses than the midline.

Often, radiology reports emphasize the most prominent abnormalities, such as spondylolisthesis but it is the other elements or another spinal level causing the stenosis that are critical in determining the best surgical approach. The axial plane also allows the physician to better locate where the sagittal slice is made so differences in sagittal canal dimension can be correlated with axial compression from facet, bone, and ligaments. Using the axial MRI plane to evaluate the cause of stenosis is important since findings such as inter-facet joint fluid, indicated by a high T2 signal within the facet joint, may be more indicative of segmental instability (Figure 7).

## Discussion

Determining the best treatment options, including surgical procedures, in patients with LSS is based on a combination of very specific clinical symptoms, the presence of neurogenic claudication and confirmatory radiologic findings. Clinically, these patients should have more leg complaints than back pain which can be confirmed with scores using the Visual Analog Pain Scale (VAS), the Oswestry Spinal Disability Index (ODI), and specifically the Zurich Claudication Questionnaire (ZCQ), as well as objective measurements of their ambulatory status [10, 16-17]. The radiologic evaluation of lumbar spinal stenosis includes both quantitative measurements in the sagittal and axial plane and qualitative descriptions, which are more subjective, using degree and percentage of stenosis as well as determining if there is segmental instability [4-5]. These two types of radiologic evaluations, qualitative and quantitative, have been compared among different specialty spine and neuro-radiology physicians with relatively consistent results when using one or the other type of evaluation [18]. The North American Spine Society (NASS) and Society of Neuroradiologists have actually published a standardized description and nomenclature for spinal disc disease and stenosis [3]. Studies have consistently demonstrated that each specialty's understanding of the nuances of the clinical findings and spinal radiology often is different, but fairly consistent within each specialty and not surprisingly with each group more accurate with the more experienced specialist [18-19]. Now, as newer physicians from interventional pain management and interventional neuroradiology are being trained to perform less invasive procedures for lumbar stenosis they need to become familiar with the complexity and nuances of CT and MRI diagnosis in LSS and how it correlates with clinical symptoms, dynamic radiographs, the use of standardized questionnaires and provocative testing used for evaluating neurogenic claudication that is associated with LSS in these patients. Physicians treating LSS must understand the range of conservative treatments, procedures and the fact that different patients with different grades of severity of stenosis and clinical symptoms may need access to different therapies correlated with their clinical symptoms rather than their radiologic findings even when there is high grade stenosis or documented spinal movement and instability. Depending on the findings, the neurosurgeon or orthopedic spine surgeon is able to choose between open decompression with instrumentation to stabilize the spine, localized decompression with interlaminar dynamic stabilization or even stand-alone interspinous stabilization. In contrast, the interventional pain physician or interventional neuroradiologist who is only trained to do minimal decompression or an interspinous stabilization needs to understand the limits of the procedures he may be trained to perform and how to use the radiologic studies and clinical findings, and questionnaires to help determine the appropriate surgical option or consult with other specialists. There are many patients where a limited or inadequate decompression or a single interspinous implant may not be able to address the entire length and volumetric stenosis and this may lead to poorer results or short term symptomatic recurrence [18-19]. There are specific issues in reviewing radiologic studies that must be considered. The relative degree of canal narrowing, especially in comparison to adjacent levels, may be a significant factor in determining whether the stenosis is clinically significant and can be treated with a minimally invasive procedure. Clinical experience has shown that often treating the most severe area of compression relieves the majority or all of the claudication symptoms. There are cases where the spondylolisthesis is at one level but the stenosis is at another level so incomplete studies could result in inadequate decompression of the stenosis or the procedure performed at the incorrect level causing symptoms of stenosis. 

**Figure 8:**
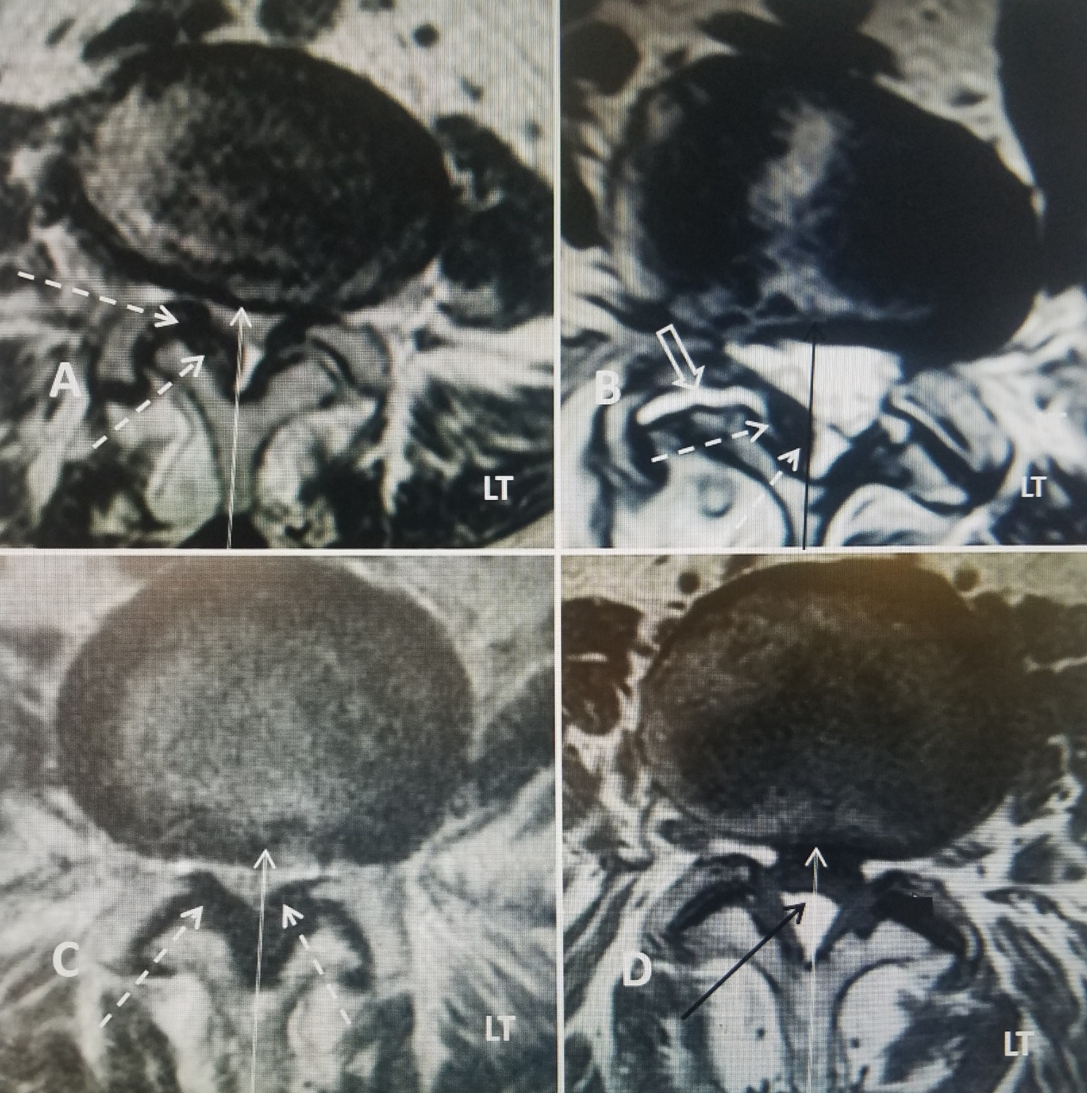
Four cases showing the axial T2 MRI at L4-5 showing different anatomic locations and severity of the stenosis from central stenosis to lateral recess stenosis. Left is marked (LT). When correlated with clinical symptoms and findings, different methods of correcting the lumbar stenosis may be indicated. A: Axial T2 MRI showing bilateral but more marked right lateral recess stenosis due to ligamentous hypertophy (dashed white arrow). The midline is marked (thin white line). B: Axial T2 MRI demonstrating right asymmetric facet hypertrophy with an enlarged right facet joint and unilateral lateral recess stenosis (dashed white arrow). There is a high T2 signal in the facet joint (open white arrow) indicating facet joint fluid, a sign of possible instability. There is additional ligamentous hypertrophy only on the right side indicated by thick black MRI signal under the lamina adjacent to the facet joint (dashed white arrows). The enlarged facet joint is encroaching on the midline (solid thin black arrow) but the left side is normal. C: Axial T2 MRI with thick symmetric ligamentous hypertrophy causing severe canal stenosis (dashed white arrows). The ligamentous hypertrophy (solid white arrows) completely encroaches up to the midline (thin white line). D: Axial T1 MRI showing bilateral symmetric facet hypertrophy. The midline is marked (thin solid white line) and there is a facet fluid cyst causing dural compression (solid black arrow).

Axial images, especially with high-quality MRI scans, are essential in establishing if the stenosis is symmetric or more in the lateral recess and neural foramina. Evaluation of lateral recess and foraminal stenosis may also require more detailed CT scans. The finding of midline narrowing compared to very localized narrowing in the lateral recess or neural foramina is important often leading to different symptoms and treatment options depending if the patient has radiculopathy verse clinical significant neurogenic claudication. Severe midline stenosis of the canal compresses the dural sac and nerve roots leading to root edema and loss of definition of the individual rootlets seen on the normal axial MRI [4-5]. A patient with more lateralized symptoms and localized foraminal stenosis may be a candidate for more localized foraminal decompression. Lateral recess and foraminal stenosis can be caused by a combination of overgrowth of the facet joint, ligamentous hypertrophy, facet fluid or facet cyst [8]. Inter-laminar and interspinous devices primarily prevent spinal extension that typically worsens neurogenic claudication but can also distract and partially open the neural foramina that relieve radicular symptoms. Severe foraminal stenosis caused by facet overgrowth may still require actual surgical decompression of the nerve root (Figure 8).

Dynamic and standing X-rays and MRI scans provide additional information. Vacuum changes seen on some plain X-rays and especially on CT scan can be a secondary indication of segmental instability. Up to 5 mm of anterolisthesis without worsening with movement is regarded as 'stable' grade one spondylolisthesis. Movement and especially alignment in extension greater than 3-5 mm or 50% of the existing listhesis is felt to be indicative of instability [7-8]. Clinical symptoms in patients with lumbar spinal stenosis are typically aggravated by standing and spinal extension, and it is hypothesized that spinal extension causes 'buckling' of the ligamentum flavum into the posterior lumbar canal, that is already anatomically narrowed, positionally aggravating the compression and symptoms [9, 11]. Standing flexion and extension MRI scans show worsening of posterior ligamentous compression with spinal extension giving support to these clinical observations [6-7]. Limiting the lumbar extension is the basic mechanism explained for the use and effectiveness of a variety of different posterior interlaminar and interspinous distraction and stabilization devices [5, 7]. The posterior wall of the neural foramina is formed by the facet joints and facet capsule and interlaminar and interspinous devices can add 2-3 mm of vertical distraction, opening the neural foramina. With spinal extension the foramina can narrow so preventing this relieves postural symptoms since the dura and nerve roots are not compressed during spinal extension [4, 7]. The current options for surgical treatment have recently expanded from lumbar decompression, decompression with pedicle screw fixation to include both limited micro laminar decompression, interlaminar decompression with interlaminar stabilization (Coflex^R^, Paradigm Spine, New York, USA), simple interspinous stabilization without decompression (Vertiflex^R^, Superion, Santa Clara, California, USA) and the (MInuteman^R^ interspinous spacer, Spinal Simplicity, Overland Park, Kansas, USA ). Published guidelines for using the posterior systems including, Vertiflex and Coflex have been based on a simple percentage reduction in canal sagittal size, i.e. less than 25% to 50% for interspinous spacers and greater than 50% stenosis for various decompression procedures with or without interspinous spacers or pedicle screws [15,16]. Other approaches, all leading to increasing the axial area of the canal include radiofrequency ligamentous ablation, minimally invasive lumbar decompression (MILD procedure) and pedicle expansion osteotomy. Radiofrequency ablation was shown to give clinical relief of claudication by shrinking stenosis secondary to hypertrophied ligament flavum or an enlarged or facet cyst [12-13]. Some reports show that tubular or minimally invasive lumbar Decompression (MILD) procedure, a mostly midline removal of the ligamentum flavum and laminar edge can also be effective in relieving neurogenic claudication from LSS [14]. Pre and post-operative axial CT scan studies with pedicle lengthening osteotomy demonstrated that by lengthening the pedicle 2-5 millimeters, which led to dorsal lengthening of the canal without lateral decompression resulted in 30% to 50% increase in axial spinal area [12]. What all these minimal approaches to LSS have in common is the ability to either increase the canal sagittal dimension, if even by a few millimeters, which in turn increases the spinal canal area, with or without limiting the extension of the lumbar spine. Volumetric changes may be more important than simple localized decompression to increase the spinal canal area [19]. Volumetric studies of four positions (supine, flexed, standing and extension) with three-dimensional CT and MRI of both volunteers with normal spinal canals and patients with symptomatic LSS with neurogenic claudication both demonstrated that the spinal canal volume was larger in the supine and flexed postures compared to standing and extension, however the spinal canal volume in symptomatic patients with LSS was significantly lower in all four postures [19].

Proper selection and technical execution of these different procedures results in more spinal area, less positional change, and less dynamic spinal canal narrowing leading to resolution of symptoms of neurogenic claudication. There have not been many detailed studies analyzing the radiology findings for selecting one procedure over the other for treating LSS. Generally a qualitative percentage or degree of sagittal stenosis has been used as the reason to choose one procedure over the other [2, 4-5]. Previously, spinal decompression with or without fixation was performed only by neurological surgeons or orthopedic spine surgeons. Less invasive procedures have been developed that in selected cases have equivalent outcomes with good five year followup, using specific radiologic and clinical criteria [11-14]. Now, these minimally invasive procedures are being taught to and performed by pain physicians, anesthesiologists and interventional neuro-radiologists. This introduces several variables since both the type and experience of the physician preoperatively analyzing the patient and specifically the radiology criteria and determining the number of spinal levels involved, the cause, degree and type of lumbar spinal stenosis present and understanding how it correlates with clinical symptoms of neurogenic claudication is changing. A comprehensive set of guidelines incorporating clinical findings, treatment steps, and broad radiologic findings and criteria for lumbar spinal stenosis was recently published for Minimally Invasive Spine Treatment (MIST) [20]. More detailed use of various spinal canal measurements, and analyzing the stenosis especially in the axial plane rather than as a simple percentage of sagittal narrowing as presented in these examples will hopefully guide and make more precise the description and the selection process for these more minimal procedures by a variety of different physicians.

## Conclusions

The interpretation and integration of information obtained from clinical testing, specifically designed lumbar stenosis questionnaires and radiologic studies are all critical in evaluating a patient with LSS. Dynamic X-rays demonstrate if there is instability even when scoliosis or spondylolisthesis is not present. Sagittal views on CT and MRI identify the levels involved while axial views show if the stenosis is central or more localized to one side and due to the soft tissue of bone compression. The degree of canal stenosis is best measured on axial films using an area measurement and then comparing it to the area at adjacent spinal levels. Actual spinal canal area can be calculated either directly from the area cursor on many scan programs or indirectly by a multiplication of the sagittal depth and the mid-facet width. The spinal canal area measurement, when compared to similar measurements at adjacent spinal segments gives a better picture of the relative degree of stenosis and if it is confined to one spinal segment or extends over multiple levels. It is clear that the midline sagittal measurements and estimates of canal narrowing, either by degree, percentage or measurement in millimeters can miss or underestimate lateral stenosis. Integrating and comparing both CT and MRI in all planes helps differentiate soft tissue ligamentous stenosis from bone overgrowth and facet hypertrophy in patients with spinal stenosis. Study of these measurements may be critical in helping to define whether the best procedure is more minimally invasive, with indirect decompression for lumbar stenosis with neurogenic claudication, or some degree of bone decompression and stabilization based on consistent radiologic follow-up CT and MRI measurements rather than percentages of reduction in canal size.
